# Cohort Differences in Physical Health and Disability in the United States and Europe

**DOI:** 10.1093/geronb/gbae113

**Published:** 2024-06-20

**Authors:** Laura Gimeno, Alice Goisis, Jennifer B Dowd, George B Ploubidis

**Affiliations:** Centre for Longitudinal Studies, Social Research Institute, Institute of Education, University College London, London, UK; Centre for Longitudinal Studies, Social Research Institute, Institute of Education, University College London, London, UK; Leverhulme Centre for Demographic Science, Nuffield Department of Population Health, Nuffield College, University of Oxford, Oxford, UK; Centre for Longitudinal Studies, Social Research Institute, Institute of Education, University College London, London, UK; (Social Sciences Section)

**Keywords:** Birth cohorts, Chronic disease, Disabilities, International comparison

## Abstract

**Objectives:**

Declines in mortality have historically been associated with improvements in physical health across generations. While life expectancy in most high-income countries continues to increase, there is evidence that younger generations, particularly in the United States, are less healthy than previous generations at the same age. We compared generational trends in physical health in the United States, England, and continental Europe to explore whether other regions have experienced a similar pattern of worsening health across cohorts.

**Methods:**

Using data from nationally representative studies of adults aged ≥50 years from the United States (Health and Retirement Study, *n =* 26,939), England (English Longitudinal Study of Ageing, *n =* 14,992) and 11 continental European countries (Survey of Health, Ageing and Retirement in Europe, *n =* 72,595), we estimated differences in the age-adjusted prevalence of self-reported chronic disease and disability and observer-measured health indicators across pseudo-birth cohorts (born <1925, 1925–1935, 1936–1945, 1946–1954, 1955–1959).

**Results:**

Age-adjusted prevalence of doctor-diagnosed chronic disease increased across successive cohorts in all regions. Trends in disability prevalence were more regionally varied. Still, in both the United States and Europe, we observed a structural break in disability trends, with declines observed in prewar cohorts slowing, stalling, or reversing for cohorts born since 1945.

**Discussion:**

In all regions, we found evidence for worsening health across cohorts, particularly for those born since 1945. While more chronic disease in younger cohorts need not necessarily translate to worse quality of life or higher rates of functional limitation, there is some suggestion that worsening chronic disease morbidity may be spilling over into worsening disability.

By 2050, nearly one fifth of the world’s population will be aged ≥65 years due to long-term declines in fertility and increasing life expectancy (LE; UN, 2020). Population aging in European countries and the United States has already reached an advanced stage, with 18.6%, 21.1%, and 16.8% of the populations of England, the European Union, and the United States aged ≥65 years by 2020/2021 (Caplan & Rabe, 2023; Eurostat, 2023; ONS, 2023). Population aging is expected to accelerate as the large Baby Boom generation enters old age. The growing number and proportion of older people in aging populations have profound societal implications, increasing demand for health and social care (Scott, 2021). These negative effects of population aging could be mitigated by continued improvements in health and functioning across generations. A key question is whether more recent generations are in better health than previous generations at the same age.

Increases in longevity have historically been tied to improvements in population health. Declines in infectious disease mortality largely drove early increases in LE during the 19th and early 20th centuries through improved sanitation, nutrition, public health (McKeown & Record, 1962), and medical innovation. Since the 1980s, the prevalence of disability and functional limitations at older ages has declined in many high-income countries (Parker & Thorslund, 2007), suggesting that health and physical functioning were improving across successive generations. While the duration or percentage of life spent with disability and limitations increased in some contexts, this was often due to gains in LE outpacing gains in disability-free LE (Spiers et al., 2021). However, a growing body of research, particularly from the United States, suggests that more recently born generations, particularly those born since 1945, may be experiencing worse health than previous generations as measured by chronic disease and disability (Freedman et al., 2013; Martin et al., 2010; Seeman et al., 2010). We refer to this trend of worsening health across cohorts at the same age as the “generational health drift.”

There is some evidence that other high-income countries may also be experiencing a generational health drift for outcomes including disability (e.g., France: Cambois et al., 2013), self-rated health (Jivraj et al., 2020; Ploubidis & Pongiglione, 2019), and chronic disease (e.g., Sweden: Gondek et al., 2021; England: Jivraj et al., 2020; The Netherlands: Oostrom et al., 2016; Spain: Walter et al., 2016). However, combining such findings to understand how generational trends in Europe directly compare to those found in the United States is challenging due to the wide variety of health outcomes studied, methods, data sources, and outcome definitions used, and age-groups and periods included in the analyses (Lafortune & Balestat, 2007). For instance, many studies explore trends in the duration of life spent in poor health, which is related to but distinct from whether more recent generations are healthier at the same age and has different implications for public health. Time spent in poor health can increase despite improvements in health at the same age, provided increases in survival are greater than postponement of disease or disability onset. While European countries share many qualities with the United States (Western, Educated, Industrialized, Rich, and Democratic), there are also important differences and distinct social, economic, political, and epidemiological histories. Knowing whether and how generational trends in health differ across regions is an important first step to identifying potential drivers of generational health drift, informing strategies to halt or reverse this process.

Comparing generational trends in health across countries using similarly collected and harmonized data could overcome some of the challenges described above, but thus far there is little work in this area. Existing studies comparing trends in health across regions using this approach focus on a limited set of outcomes: disability, a single (and severe) form of poor physical health (Chatterji et al., 2015; Fors et al., 2022; Lee et al., 2020; Welsh et al., 2021), and cardiometabolic diseases (Martinson et al., 2022). Some research does suggest that trends in disability might be diverging at younger and older ages, consistent with the idea of generational drift, but trends in chronic disease and disability have not been explored together in the same study. Considering a broader set of physical health outcomes capturing different aspects of population health can contribute to a better understanding of the factors driving the generational health drift and the societal implications of these trends (e.g., for the labor market, and demand for different areas of health and social care, thereby informing funding allocation across these services; Parker & Thorslund, 2007).

In this study, we explore generational differences in physical health across cohorts born before and after the Second World War in five high-income regions: the United States, England, and Western, Northern, and Southern Europe (WE, NE, and SE). Using harmonized data from the Gateway to Global Aging Data, we explore trends for a variety of physical health outcomes, including self-reported doctor-diagnosed chronic disease, disability, and other health indicators. We directly compare these generational trends to explore whether English and European populations have experienced a similar pattern of “generational health drift” as the United States across multiple measures of health.

## Method

### Study Participants

We used data on adults aged ≥51 years from the United States, and ≥50 years from England and 11 continental European countries (Austria, Belgium, Denmark, France, Germany, Greece, Italy, The Netherlands, Spain, Sweden, and Switzerland) who participated in the Health and Retirement Study (HRS), the English Longitudinal Study of Ageing (ELSA), or the Survey of Health, Ageing, and Retirement in Europe (SHARE) between 2004 and 2018. HRS, ELSA, and SHARE have similar target populations, frequency and timing of interviews (every 2 years, except SHARE in 2008 when no health-related outcomes were collected), multidisciplinary questionnaires, and use of refreshment samples to maintain sample representativeness (Börsch-Supan et al., 2013; Sonnega et al., 2014; Steptoe et al., 2013). We used harmonized datasets produced by the Gateway to Global Aging Data: Harmonized HRS Version C, Harmonized ELSA Version G2, and Harmonized SHARE Version F (Chen et al., 2022; Wilkens et al., 2021, 2022).

The analytical sample was comprised of repeated observations of respondents from HRS, ELSA, and SHARE between 2004 and 2018 who were age-eligible and had a known year of birth. Proxy responses were included where available (Supplementary Figures 1–3).

HRS received ethical approval from the University of Michigan Health Sciences/Behavioural Sciences Institutional Review Board, ELSA from the South Central–Berkshire Research Ethics Committee, and SHARE from the Ethics Council of the Max Planck Society and the University of Mannheim. All participants gave informed consent. No further ethics approval was required for this work.

### Exposure

The pseudo-birth cohort approach allows for flexible use of longitudinal data by treating each wave as a cross-sectional sample and considering respondents at each time point to be representative of those in their birth cohort who are alive at a given age. We grouped respondents into five pseudo-birth cohorts, defined by birth year, roughly corresponding to the Greatest Generation (born before 1925), the early Silent Generation (born 1925–1935), the late Silent Generation (1936–1945), early Baby Boomers (1946–1955), and late Baby Boomers (1955–1959; Supplementary Figure 4). We used these groupings as we wanted to explore whether generational trends differed for those born before or after the Second World War. Each pseudo-birth cohort consisted of multiple observations of respondents within each birth year group at different ages. To be included in the sample, respondents had to have survived until at least 2004, resulting in members of earlier-born pseudo-cohorts first being observed at older ages than those of later-born pseudo-cohorts.

### Outcomes

We explored trends for self-reported and observer-measured outcomes capturing diverse aspects of physical health: self-reported doctor-diagnosed disease, disability and physical functioning, and other health indicators (self-reported and measured). Additional information on how these outcomes were measured can be found in Supplementary Table 1.

Participants were asked if they had ever been told by a doctor that they had cancer (excluding minor skin cancers), heart problems, chronic lung disease (e.g., chronic bronchitis or emphysema), high blood pressure (BP), or high cholesterol (for ELSA and SHARE). Each outcome was a binary indicator (yes/no) of whether a participant reported having each condition at each wave they participated in.

Participants were also asked about difficulties with six activities of daily living (ADLs) related to personal care activities (e.g., eating, bathing), four instrumental activities of daily living (IADLs) related to daily tasks (e.g., preparing a hot meal, shopping for groceries), and their ability to perform seven mobility and motor coordination tasks (e.g., walking one block, lifting 10 lbs, picking up a small coin from a flat surface). Using answers to these questions, we constructed four binary indicators to identify respondents with isolated mobility limitations only (≥1 mobility difficulty but no IADL or ADL difficulties), mild disability (≥1 IADL difficulty and any number of mobility difficulties but no ADL difficulties), moderate disability (1–2 ADL difficulties and any number of IADL or mobility difficulties), or severe disability (≥3 ADL difficulties and any number of IADL or mobility difficulties) at each wave. These categories were constructed to reflect the idea that mobility limitations do not inevitably result in the inability to perform specific activities, but that there is a high correlation between mobility limitation, IADL limitation, and ADL limitation: a person with ADL limitations is likely to also have IADL and mobility limitations.

Changes in screening and clinical definitions and thresholds used to diagnose disease may result in members of more recent cohorts being more likely to receive a diagnosis, complicating measurement of underlying health. We therefore also explored trends in observer-measured outcomes, to understand whether cohort trends reflect changes in diagnosis versus true changes in objective health. We explored generational trends in continuous maximum grip strength (in kilograms) and binary indicators of obesity (self-reported body mass index [BMI] ≥30 kg/m^2^ for HRS and SHARE and measured BMI ≥30 kg/m^2^ for HRS and ELSA) and measured hypertension (mean systolic BP ≥140 mmHg or mean diastolic BP ≥90 mmHg for HRS and ELSA). While not direct measurements of disease, these are indicators of physical health status correlated with disease and disability risk, and act as useful markers of potential disease or disability risk, which can be captured earlier in life.

### Covariates

Covariates in all models were age (year of interview minus birth year) and gender (man or woman). In models for grip strength, we additionally adjusted for height (in meters) and weight (in kilograms) because grip strength is associated with body size. Calendar year was not included as a covariate because our intent was not to isolate a “cohort effect” but rather to describe differences in disease and disability prevalence accounting for age between generations regardless of whether these resulted from period or cohort effects.

### Data Analysis

We used a pseudo-cohort approach, treating each wave as a cross-sectional sample. We tested the association between birth cohort and each outcome of interest using modified Poisson regression with robust standard errors for binary outcomes (adjusting for age, age^2^, and gender), which has the advantage of producing risk ratios (RRs) that are more intuitive to interpret than odds ratios (Zou, 2004; Zou & Donner, 2013), and avoids issues of noncollapsibility of the odds ratio (Pang et al., 2016). We explored trends for grip strength using linear regression (adjusting for age, age^2^, gender, height, and weight). RRs were estimated for each country separately using the 1936–1945 cohort as reference group, because this group had the most overlap in age with other pseudo-cohorts, and was one of the largest pseudo-cohorts. We accounted for survey design and clustering of observations within individuals in calculating the standard errors, and used cross-sectional weights and inverse-probability weights for nonresponse (more details are given below and in the Supplementary Material). We present trends in five regions: the United States, England, WE (Austria, Belgium, France, Germany, The Netherlands, and Switzerland), NE (Sweden and Denmark), and SE (Spain, Italy, and Greece). Estimates for countries in continental European regions were statistically pooled using random-effects meta-analysis. Data were analyzed using Stata (Version 18; StataCorp LP, College Station, TX).

### Missing Data Strategy

Owing to their longitudinal design, HRS, ELSA, and SHARE are affected by attrition, which varies by sociodemographic and health status. Even when the data are used cross-sectionally, as we do in this study, results may be biased as the sample’s composition at each cross-section is affected by attrition in the longitudinal sample. We derived inverse-probability weights for nonresponse at each wave to mitigate the impact of differential nonresponse. These weights were multiplied with person-level cross-sectional weights provided by the studies, which restore representativeness by gender and age/cohort, to obtain a final analysis weight. We describe the derivation of these weights in the Supplementary Material.

### Sensitivity Analyses

We examined results stratified by gender. We also explored how results differed when using cross-sectional weights only, and when additionally adjusting for the total number of waves participants responded to. Using biomarker data from ELSA, we explored generational differences in glycated hemoglobin (HbA1c, a marker of clinical diabetes), total cholesterol, and BP levels accounting for medication. Details are provided in the Supplementary Material.

## Results

The analytical sample was composed of 435,069 observations of 114,526 participants in HRS, ELSA, and SHARE who participated in at least one survey wave between 2004 and 2018 and were age-eligible at the time of interview (Table 1). Birth year ranged from 1896 to 1959, with a mean birth year of 1943 (*SD* = 10 years). The mean number of waves was 3.8, with members of the 1936–1945 cohort participating in the most waves on average (Supplementary Table 2). Item nonresponse was low, though the prevalence of missing data was higher for observer-measured outcomes in all three studies (Supplementary Tables 3–5).

**Table 1.** Sociodemographic and Health Characteristics of the Analytical Sample (*n* = 114,526) by Birth Cohort

Variables	Birth cohort					Total
	<1925	1925–1935	1936–1945	1946–1954	1955–1959	
	*N* (%/mean, *SD*)	*N* (%/mean, *SD*)	*N* (%/mean, *SD*)	*N* (%/mean, *SD*)	*N* (%/mean, *SD*)	*N* (%/mean, *SD*)
Total number	8,423 (100)	22,223 (100)	31,547 (100)	35,922 (100)	16,411 (100)	114,526 (100)
Gender						
Man	3,150 (37)	10,275 (46)	14,963 (47)	16,886 (47)	7,117 (43)	52,391 (46)
Woman	5,273 (63)	11,948 (54)	16,584 (53)	19,036 (53)	9,294 (57)	62,135 (54)
Region						
United States	3,345 (40)	5,792 (26)	6,747 (21)	6,731 (19)	4,324 (26)	26,939 (23)
England	1,220 (14)	2,921 (13)	4,111 (13)	5,022 (14)	1,718 (11)	14,992 (13)
Western Europe	2,022 (24)	7,123 (32)	11,198 (35)	13,589 (38)	5,928 (36)	39,860 (35)
Northern Europe	667 (8)	2,020 (9)	3,435 (11)	3,774 (10)	1,411 (9)	11,317 (10)
Southern Europe	1,159 (14)	4,367 (20)	6,056 (20)	6,806 (19)	3,030 (18)	21,418 (19)
Education						
Degree	851 (10)	2,991 (14)	5,917 (19)	8,855 (25)	4,363 (27)	22,977 (20)
No degree	7,457 (90)	18,931 (86)	25,278 (81)	26,677 (75)	11,938 (73)	90,281 (80)
Doctor diagnoses						
Cancer	1,472 (18)	4,214 (19)	5,502 (17)	4,095 (11)	1,344 (8)	16,627 (15)
Heart problems	3,664 (44)	8,449 (38)	8,574 (27)	5,847 (16)	1,840 (11)	28,374 (25)
Lung disease	1,149 (14)	3,423 (15)	4,174 (13)	3,313 (9)	1,317 (8)	13,376 (12)
Diabetes	1,539 (18)	5,308 (24)	6,930 (22)	6,108 (17)	2,388 (15)	22,273 (19)
High blood pressure	5,038 (60)	14,248 (64)	18,848 (60)	17,264 (48)	6,609 (40)	62,007 (54)
High cholesterol	1,404 (25)	8,342 (43)	13,499 (46)	13,894 (40)	5,560 (35)	42,699 (40)
Disability						
Mobility limitation	3,963 (47)	14,745 (66)	20,326 (65)	19,870 (56)	8,013 (50)	66,917 (59)
Mild disability	1,932 (23)	4,024 (18)	2,964 (9)	2,242 (6)	1,008 (6)	12,170 (11)
Moderate disability	3,659 (43)	7,625 (35)	7,325 (23)	5,778 (16)	2,292 (14)	26,829 (23)
Severe disability	2,955 (35)	4,321 (19)	2,774 (9)	2,045 (6)	815 (5)	12,910 (11)
Biomarkers						
Obesity (measured)^a^	533 (23)	2,677 (40)	4,015 (45)	4,618 (49)	2,455 (51)	14,298 (44)
Obesity (self-reported)^b^	1,054 (15)	5,044 (27)	8,348 (31)	9,466 (31)	4,667 (32)	28,579 (29)
Hypertension^a^	1,064 (43)	3,699 (54)	5,020 (55)	4,675 (48)	1,988 (40)	24,125 (73)
Maximum grip strength (kg)^c^	5,733 (23.0, 8.9)	19,232 (29.1, 10.4)	28,840 (34.4, 11.3)	33,109 (37.9, 12.0)	14,919 (38.5, 12.1)	101,833 (34.7, 12.2)

*Notes*: ADL = activities of daily living; ELSA = English Longitudinal Study of Ageing; HRS = Health and Retirement Study; IADL = instrumental activities of daily living; SHARE = Survey of Health, Ageing, and Retirement in Europe. Percentages, means, and standard deviations (*SD*s) are unweighted. Values correspond to the number of individuals who reported each outcome at least once between 2004 and 2018. Mobility limitation = difficulty with ≥1 mobility task but no IADL or ADL limitation. Mild disability = difficulty with ≥1 IADL but no ADL limitation. Moderate disability = difficulty with 1 or 2 ADL. Severe disability = difficulty with ≥3 ADL. Some individuals had no instances where the outcome was recorded. A description of item nonresponse is given in Supplementary Tables 2–4.

^a^Only measured in HRS (United States) and ELSA (England).

^b^Only measured in HRS (United States) and SHARE (continental Europe).

^c^Mean of the maximum grip strength recorded for each participant across all waves.

### Main Results

#### Self-reported doctor-diagnosed chronic diseases

**Figure 1.**
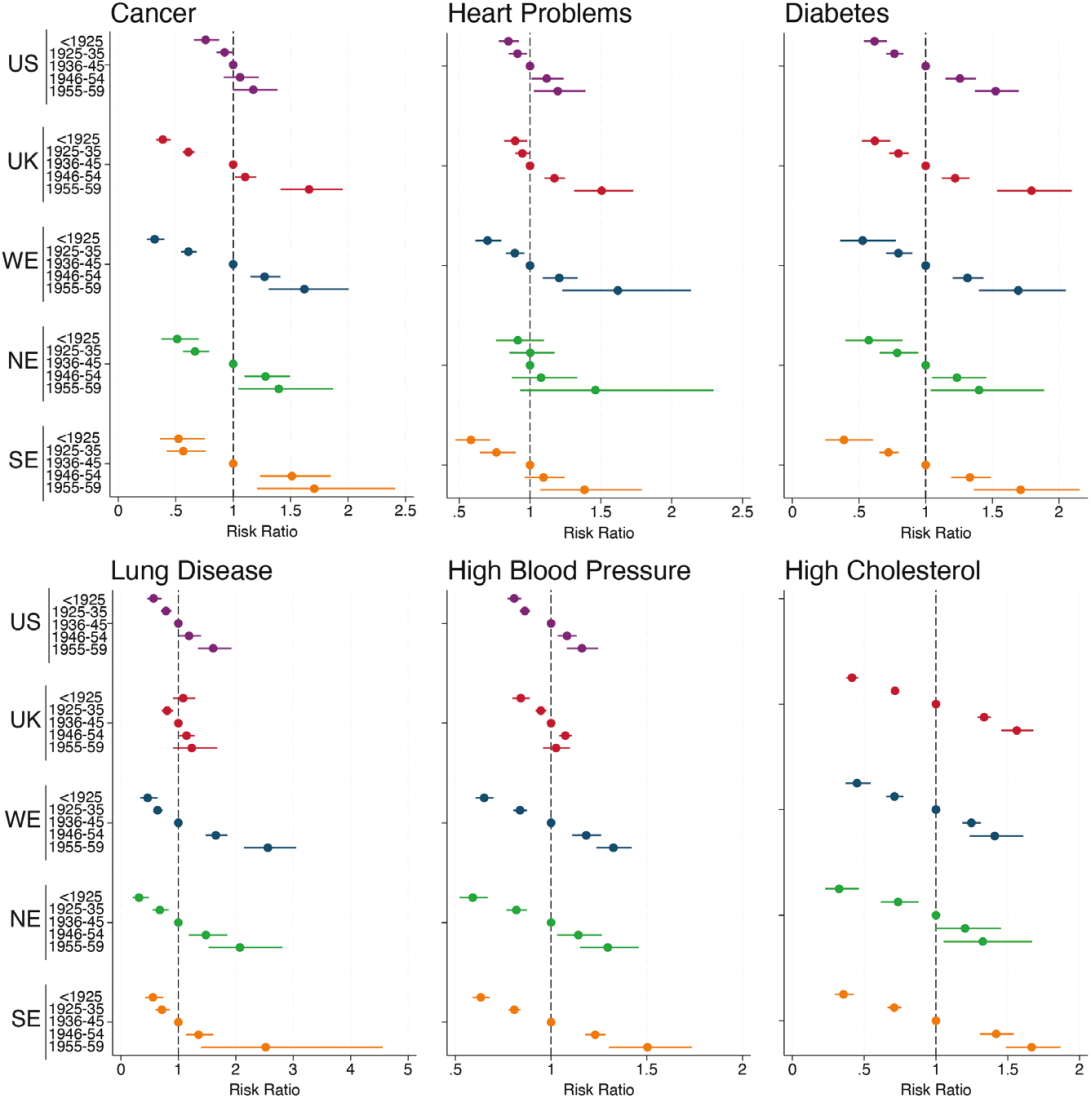
Generational differences in self-reported doctor-diagnosed chronic disease in the United States and Europe. NE = Northern Europe; SE = Southern Europe; UK = United Kingdom; US = United States; WE = Western Europe. Reference group is the 1936–1945 birth cohort. All results are adjusted for age, age^2^, and gender. Results for the United States are not shown for high cholesterol, as information on this outcome was only collected in three sweeps of HRS as opposed to seven in ELSA and SHARE (Supplementary Table 1). A risk ratio (RR) >1 indicates that a cohort has a greater prevalence of the outcome than the reference 1936–1945 cohort accounting for age and gender, while an RR <1 indicates that the cohort had a lower prevalence. ELSA = English Longitudinal Study of Ageing; HRS = Health and Retirement Study; SHARE = Survey of Health, Ageing, and Retirement in Europe.

Overall, the age- and gender-adjusted prevalence of doctor-diagnosed chronic disease increased across successive cohorts in the United States and Europe (Figure 1). The prevalence of diabetes and high cholesterol increased in all five regions. Generational trends in cancer, high BP, lung disease, and heart problems also suggested that more recently born cohorts had worse health at the same age. However, there was more regional variation in the magnitude of RRs and the specific cohorts with the largest RRs. For instance, RRs were systematically larger in SE compared to the United States for all six health outcomes, suggesting that prevalence had increased more across cohorts in SE than the United States. For cancer, lung disease, and heart problems, which are outcomes capturing many specific conditions, 95% confidence intervals were wider than for specific diagnoses such as diabetes. Still, the overall direction of generational trends was consistent across regions.

#### Disability and mobility limitation

**Figure 2.**
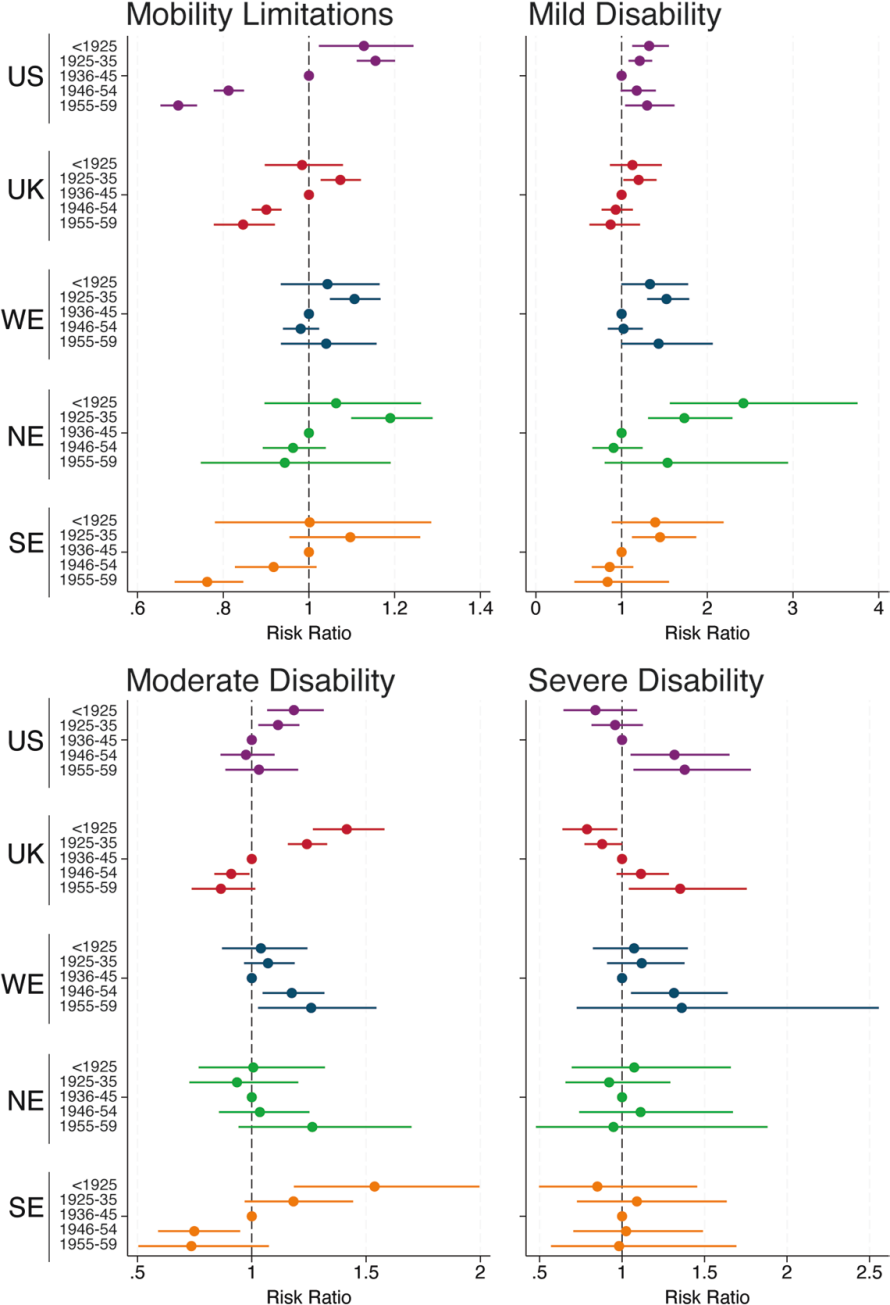
Generational differences in self-reported disability and mobility limitation in the United States and Europe. NE = Northern Europe; SE = Southern Europe; UK = United Kingdom; US = United States; WE = Western Europe. Reference group is the 1936–1945 birth cohort. All results are adjusted for age, age^2^, and gender. Mobility limitations = respondent reports ≥1 mobility limitation, but no IADL or ADL limitations. Mild disability = respondent reports ≥1 IADL limitation and any number of mobility limitations but no ADL limitations. Moderate disability = respondent reports 1–2 ADL limitations, and any number of IADL or mobility limitations. Severe disability = respondent reports ≥3 ADL limitations, and any number of IADL or mobility limitations. A risk ratio (RR) >1 indicates that a cohort has a greater prevalence of the outcome than the reference 1936–1945 cohort accounting for age and gender, while an RR <1 indicates that the cohort had a lower prevalence. ADL = activities of daily living; IADL = instrumental activities of daily living.

Trends in disability and mobility limitation were more regionally varied (Figure 2); however, in the United States and most European regions, declines in disability prevalence seen in prewar cohorts appeared to slow, stall, or reverse in postwar cohorts. In the United States, the age-adjusted prevalence of isolated mobility limitations (without IADLs or ADLs) declined across cohorts, beginning with the 1925–1936 cohort. There was also evidence for declines in the prevalence of mild disability (IADL without ADLs) and moderate disability (1–2 ADLs) across prewar cohorts, but the prevalence remained stable or increased again in cohorts born after 1945. The prevalence of severe disability (≥3 ADLs) increased in postwar cohorts. Trends in England were similar, though the prevalence of mild disability remained stable across postwar cohorts rather than reversed and declines in prevalence of moderate disability slowed but did not stall entirely. The shape and direction of generational trends in SE were very similar to those in England, except for severe disability, for which we found no strong evidence of a generational trend. Despite some improvements across prewar cohorts, there was no strong evidence for further declines in age-adjusted prevalence of mobility limitations or disability across postwar cohorts in WE or NE.

#### Other indicators of physical health

**Figure 3.**
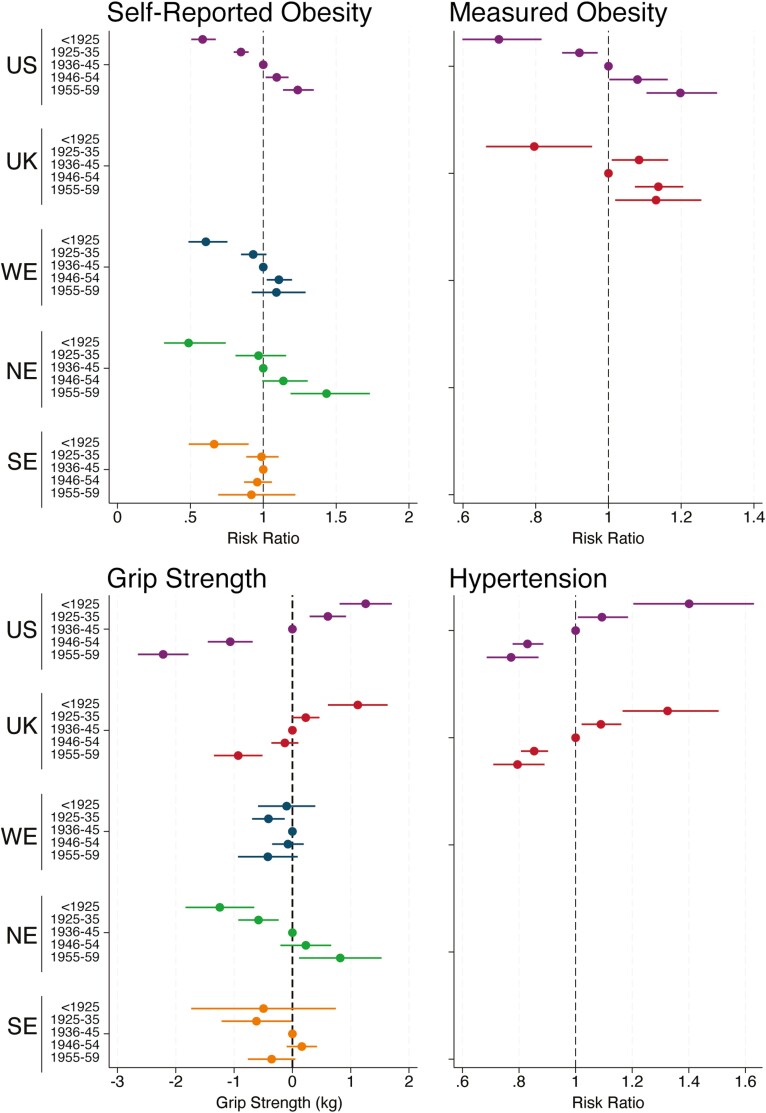
Generational differences in obesity, hypertension, and grip strength in the United States and Europe. NE = Northern Europe; SE = Southern Europe; UK = United Kingdom; US = United States; WE = Western Europe. Reference group is the 1936–1945 birth cohort. Results for obesity and hypertension are adjusted for age, age^2^, and gender. Results for grip strength are adjusted for age, age^2^, gender, height (in meters), and mass (in kilograms). Results for hypertension and measured obesity are presented for the United States and England only. Results for self-reported obesity are shown for the United States and continental European regions only. Hypertension = average systolic blood pressure ≥140 mmHg or average diastolic blood pressure ≥90 mmHg. Obesity = measured or self-reported BMI ≥30 kg/m^2^. Grip strength (kilograms) is a continuous outcome. A risk ratio (RR) >1 indicates that a cohort has a greater prevalence of the outcome than the reference 1936–1945 cohort accounting for age and gender, while an RR <1 indicates that the cohort had a lower prevalence. For grip strength, a point estimate >0 is indicative of stronger grip strength in a cohort compared to the reference 1936–1945 reference cohort, while a point estimate <0 is indicative of weaker grip strength accounting for age, gender, height, and weight. BMI = body mass index.

Age-adjusted obesity prevalence increased across cohorts, based on self-reported BMI for the United States and continental European regions and measured BMI for England and the United States (Figure 3). However, in SE such increases in prevalence were only observed for cohorts born before 1945, and prevalence did not increase further in postwar cohorts. The risk of measured hypertension decreased across cohorts in both the United States and England. Trends in age, height, and weight-adjusted maximum grip strength exhibited a high degree of regional variability, with evidence for declines in the United States and England, and increases in NE across the full set of cohorts. Estimates for SE and WE showed no clear pattern, though there was some suggestion of declining grip strength in the most recently born cohorts in both regions.

### Sensitivity Analyses

Results were similar when stratifying by gender and did not differ significantly when cross-sectional weights only were used or when number of waves participated in was adjusted for (results available upon request). In ELSA, accounting for medication, more recent generations had a higher age-adjusted prevalence of high HbA1c, high total cholesterol, and hypertension (Supplementary Figure 5).

## Discussion

We compared generational trends in physical health and disability for cohorts born before and after the Second World War in the United States and Europe to explore whether a similar “generational health drift” was experienced across regions, using harmonized data from studies with consistent designs. More recently born cohorts in all regions had a higher prevalence of doctor-diagnosed chronic disease than their predecessors at the same age. Trends in functional limitation were more nuanced and regionally variable. While the age-adjusted prevalence of isolated mobility limitations declined across cohorts in most regions, declines in disability prevalence seen in prewar cohorts appeared to slow, stall, or reverse for cohorts born after 1945 in the United States and most European regions. These findings support the idea that a generational drift in physical health has occurred in both the United States and Europe, whereby younger generations have worse health than previous generations at the same age, particularly for cohorts born since 1945.

Our study used harmonized data from studies with consistent methodologies to compare generational trends across regions, and our results were largely consistent with the findings from other single-country studies. Such studies have also found that the prevalence of doctor-diagnosed disease has increased across successive cohorts. Studies on older adults in the United States have found evidence for the increasing prevalence of multimorbidity (Bishop et al., 2022), as well as of specific conditions such as cancer, diabetes, hypertension, lung disease, and heart disease (Beltrán-Sánchez et al., 2016; Payne, 2022), both within age-groups over time and across cohorts. Similarly, the age-standardized period prevalence of many major noncommunicable diseases (e.g., diabetes, lung cancer, stroke) in the United Kingdom has been increasing since 1946 (Gondek et al., 2019), and a higher risk of chronic disease morbidity has been noted across English postwar cohorts (Jivraj et al., 2020). Worsening chronic disease morbidity across cohorts has also been described in continental European countries (e.g., Spain: Walter et al., 2016; Sweden: Gondek et al., 2021; The Netherlands: Oostrom et al., 2016). We noted that RRs in the United States were often smaller than for other regions, which may be explained by changes in dietary, occupational, and smoking patterns beginning earlier in the United States, resulting in less pronounced changes in exposure to disease risk factors across these U.S. cohorts. For instance, male lung cancer mortality, attributable to smoking, began to decline for post-1920s cohorts in the United States (Preston & Wang, 2006), but only for post-1950s cohorts in southern Spain (Córdoba-Doña et al., 2023).

The regional variability in generational trends in disability and mobility limitations noted in our study has also been reported for period trends in disability prevalence (Lee et al., 2020). While the prevalence of isolated mobility limitations declined in most regions, trends in disability prevalence in several regions appeared to show a structural break around 1945, declining across prewar cohorts, but stalling or reversing in postwar cohorts. Examining trends in the prevalence of limitations affecting major activity and work in Americans aged 30–39 years born 1915–1959 (similar to the birth years included in this study), Reynolds and colleagues (1998) found that while cohorts born before 1945 progressively became less disabled, prevalence increased again for cohorts born after 1945, suggesting that the generational trends observed in our study among adults aged ≥50 years may already have been apparent in earlier life. Diverging trends in disability at younger and older ages in the United States have also been reported between 1988/1994–1999/2004 and 2000–2008 (Freedman et al., 2013; Seeman et al., 2010). In England, an absence of declines in disability in postwar cohorts of working-age adults or diverging trends in disability at younger and older ages consistent with our results have also been noted by Welsh and colleagues (2021). Comparing our results to other studies on trends in disability in continental Europe is challenging given the variety of time periods and age-groups covered in the literature. While studies on older adults (aged ≥65 years) often document declines in IADL, ADL, or mobility limitations up to the 2000s (e.g., Norway: Moe & Hagen, 2011; Spain: Zunzunegui et al., 2006), a more recent study capturing postwar cohorts who have entered older age is consistent with our findings of stalling improvements or worsening disability and functional limitation across postwar cohorts (Fors et al., 2022). The generational drift in disability could be explained by many factors, including changing associations between disability and chronic disease morbidity, multimorbidity, and obesity (Chang et al., 2017; Sperlich et al., 2021). Contextual differences in the treatment and management of these chronic conditions and in educational and occupational changes across cohorts may explain regional differences in disability trends.

Trends in other health indicators can help to contextualize trends in self-reported outcomes. The increase in age-adjusted prevalence of obesity, a risk factor for cardiometabolic disease, is consistent with increasing prevalence of outcomes such as diabetes across cohorts. We found that age-adjusted prevalence of obesity remained stable for those born after 1936 in SE, which could be explained by the compensatory effect of rapidly increasing stature across these cohorts (Garcia & Quintana-Domeque, 2007). Improvements in nutrition driving changes in childhood growth patterns and adult stature could also explain continued increases in grip strength across postwar cohorts (Kuh et al., 2006), and the lack of worsening disability in SE. Trends in grip strength by region were broadly consistent with findings from single-country studies from countries within those regions. For instance, grip strength improved across cohorts of Finnish adults aged ≥75 years born between the 1910s and 1940s, consistent with our findings for NE (Koivunen et al., 2021). In age–period–cohort models, Beller and colleagues (2019) found evidence that grip strength remained stable across birth cohorts in Sweden accounting for age and period, while Germans born 1930–1945 had stronger grip strength than previous and subsequent generations, and Spaniards experienced increasing grip strength across cohorts born up to the 1960s, at which point grip strength declined. These patterns are consistent with our findings because we did not adjust for period. Such regional differences likely reflect differences in the balance of nutritional improvements and declines in physical activity (Dodds et al., 2013), related to changes in occupation. Because lower grip strength in midlife is associated with higher risk of disability and mobility limitation in later life (Soysal et al., 2021), declining grip strength across U.S. and English birth cohorts suggests that younger generations may reach older ages in a frailer state and at higher risk of functional impairment.

To understand the possible implications of these generational trends in health, it is important to know whether worse self-reported health at the same age in younger generations reflects truly worse underlying health. Trends in self-reported outcomes can result from several mechanisms including changes in medical and diagnosis practices, changes in health awareness and reporting style across generations (e.g., due to decreasing stigma in reporting certain health conditions), and true worsening health. While untangling the contribution of these factors is complex, triangulating evidence from self-reported and measured outcomes using a consistent methodological approach, as we have done in this study, can give insight into whether the generational drift in health can be explained away by phenomena other than worsening underlying health.

We illustrate this using the example of high BP in England. The prevalence of self-reported doctor-diagnosed high BP increased across successive cohorts, although increases decelerated across postwar cohorts, possibly because of opposing trends in obesity and smoking. Policies incentivizing BP monitoring in primary care (e.g., Quality and Outcomes Framework) and screening programs (e.g., the National Health Service Health Check) may result in more people with undiagnosed high BP receiving a diagnosis of hypertension, such that individuals with high BP born more recently likely receive diagnoses earlier in life. The prevalence of measured hypertension decreased across cohorts, but after accounting for medication use the generational trends in measured hypertension were consistent with those for doctor-diagnosed high BP (Supplementary Figure 5). Biomarker measures in survey data can be affected by measurement error and selection bias (because not all participants consent to nurse visits) but are not affected by changes in health awareness or demand for diagnosis (Ploubidis & Grundy, 2011). Triangulating evidence from three BP outcomes therefore suggests that in England, younger generations have higher BP than previous generations at the same age, but that their BP is better managed, which may lower risk of more lethal and disabling conditions like cardiovascular and cerebrovascular disease.

Supplementary analyses also showed that more recent generations of older adults in England had a greater prevalence of high HbA1c and total cholesterol, consistent with increasing prevalence of doctor-diagnosed diabetes and high cholesterol in more recent generations (Supplementary Figure 5). Zheng and Echave (2021) have similarly found evidence for greater physiological dysregulation across cohorts of U.S. adults born since 1945. These findings support that, while the impact of changing diagnosis, health awareness, and reporting style cannot be ruled out, generational trends in doctor-diagnosed chronic conditions such as diabetes and high BP do reflect a true generational health drift.

While our measures of disability and mobility rely on self-reports, we do not think it likely that the stalling or reversing declines in disability in most regions can be entirely explained through changing reporting styles. Difficulty performing IADLs and ADLs is a function of both physical capability and the environment (e.g., access to technology). Environmental changes are likely to have led to less difficulty performing tasks, particularly IADLs, such that younger cohorts are likely to underreport difficulty for a similar underlying degree of impairment. An exception to this may be changes in intergenerational cohabitation, with more recent cohorts of older adults having to perform more of these tasks without support resulting in more difficulties being reported. Mobility limitations are measured by asking respondents to report whether they can perform specific physical actions, and it is unlikely that there would be systematic generational differences in how these questions are answered.

Age-specific mortality rates, particularly in earlier life, declined rapidly during the 20th century, such that the likelihood of individuals with chronic morbidity or at higher risk of chronic morbidity surviving to older ages has increased across successive cohorts. Changing survival across cohorts constitutes one possible mechanism underpinning the generational health drift, alongside true worsening health, and the magnitude of its contribution to observed trends likely varies across cohorts, being strongest in earlier-born cohorts who experienced the most dramatic declines in mortality rates at younger ages across their lifetimes. Comparisons of physical and mental health across postwar cohorts in Britain in early and midlife, when mortality is low, reveal a similar pattern of generational drift (Gondek et al., 2020; Johnson et al., 2015), suggesting that these cohort trends reflect true worsening health and are not only a consequence of changing mortality patterns.

Our work does not explicitly relate trends in physical health with trends in mortality. However, because the earliest birth cohorts included in this study now have few survivors and the latest birth cohorts have already reached their 60s, we can hypothesize about what trends in age-adjusted prevalence of poor health may mean for length of life in poor health. Provided cohort LE continues to increase or remains stable, worse health in younger generations at the same age will result in more years spent with poor physical health, driven both by continued increases in survival and by declining age of onset of morbidity, in contradiction with the compression of morbidity hypothesis (Fries, 1980). This interpretation should be made with care, because further increases in cohort LE for the most recent birth cohorts included in our analysis are uncertain. In the United States, there is some suggestion that worsening health in younger cohorts is affecting LE. Slowing or stalling declines in cardiometabolic disease-related mortality for cohorts born since the 1950s have contributed to the worsening all-cause mortality rates among middle-aged White Americans alongside increases in drug-related deaths (Masters et al., 2018). Stalling LE has also been observed in England and Wales, predating the coronavirus disease pandemic which began in March 2020 (Hiam et al., 2018). If the generational health drift does result in more years spent in poor health, this will have considerable implications for health and social care expenditure and for how funding is allocated across these services. Worsening health in younger cohorts at the same age also has important employment policy implications, as national governments move to raise the State Pension Age to respond to the challenge of population aging.

Strengths of this study include its use of large-scale harmonized data from multiple countries, of a standardized methodological approach, and that we explicitly addressed the potential impact of selection bias on cross-sectional samples by developing inverse-probability weights for nonresponse. Another strength is the wide range of health outcomes explored, allowing findings to be triangulated for a better understanding of the potential societal implications of generational trends in physical health. This study also has limitations. While HRS, ELSA, and SHARE follow similar designs, some variability between studies remains (e.g., sampling frame, timing of refreshment samples, not all outcomes measured at all waves). Our use of harmonized data meant that some outcomes (heart problems, lung disease, cancer) were broad, and included conditions which may be evolving in different directions, making the interpretation of these trends challenging. Finally, pseudo-cohorts were not observed at perfectly overlapping ages. Because members of earlier-born pseudo-cohorts were first observed at older ages, these individuals may constitute a more health-selected subset of their cohort than members of other pseudo-cohorts first observed at younger ages, resulting in a potential underestimation of poor health in older cohorts compared to the 1936–1945 reference group. However, comparisons at overlapping ages were consistent with our results, and the direction of trends across prewar cohorts in our study reflects findings from other studies comparing health across cohorts born in the first half of the 20th century. Comparisons of health across postwar cohorts are less likely to be affected by this issue because members of these pseudo-cohorts were first observed in their 50s. The lack of complete overlap in age requires some assumptions to be made about similarly shaped age trajectories in health across cohorts. Most outcomes in this study were absorbing states, for which it was not possible for respondents to transition from a “diagnosed” to an “undiagnosed” state. This was not the case for all outcomes (e.g., obesity, disability), but several studies comparing true birth cohorts in Britain have found that age trajectories of obesity and other outcomes like poor mental health are similar across postwar cohorts (Gondek et al., 2020; Johnson et al., 2015). Estimates of cohort differences in health will improve as HRS, SHARE, and ELSA continue to collect data on cohorts at overlapping ages.

Our results suggest that populations in the United States and Europe may be experiencing a generational health drift, with younger generations exhibiting worse health than previous generations at the same age for a variety of physical health outcomes, particularly for cohorts born after 1945. This drift was observed in all regions for chronic disease, while evidence for generational drift in disability was more regionally varied. Worsening quality of life and increasing disability and functional limitation are not inevitable consequences of increasing chronic disease in younger generations, provided these conditions are well-managed. Concerningly, our results suggest that previous declines in disability have stalled or reversed in the United States and some European regions, suggesting that despite improvements in medical knowledge and technology, worsening chronic disease status in these younger cohorts may be spilling over to disability. More research is needed to better understand the drivers of the generational health drift, which may vary by context. Recent work by Zheng and colleagues (2023), using a life-course framework, has highlighted the contribution of increasing obesity, job insecurity, and disease in early life to worsening health in postwar cohorts in the United States. More research using a life-course approach in different countries will contribute to a better understanding of whether these drivers are context-specific. Exploring when in the life-course generational differences in physical and mental health emerge can further contribute to better understanding mechanisms underpinning the generational health drift and, crucially, inform strategies to reverse it.

## Supplementary Material

gbae113_suppl_Supplementary_Materials

## Data Availability

All data used in this study are publicly accessible to researchers. Harmonized data sets for HRS, ELSA, and SHARE, and accompanying codebooks can be accessed via the Gateway to Global Aging Data website (www.g2gad.org). The additional HRS data used in this study can be accessed through the HRS website (www.hrsdata.isr.umich.edu).

## References

[CIT0001] Beller, J., Miething, A., Regidor, E., Lostao, L., Epping, J., & Geyer, S. (2019). Trends in grip strength: Age, period, and cohort effects on grip strength in older adults from Germany, Sweden, and Spain. SSM—Population Health, 9, 100456. 10.1016/j.ssmph.2019.100456 31453311 PMC6700453

[CIT0002] Beltrán-Sánchez, H., Jiménez, M. P., & Subramanian, S. V. (2016). Assessing morbidity compression in two cohorts from the Health and Retirement Study. Journal of Epidemiology and Community Health, 70(10), 1011–1016. 10.1136/jech-2015-206722 27103663 PMC5486403

[CIT0003] Bishop, N. J., Haas, S. A., & Quiñones, A. R. (2022). Cohort trends in the burden of multiple chronic conditions among aging U.S. adults. The Journals of Gerontology, Series B: Psychological Sciences and Social Sciences, 77(10), 1867–1879. 10.1093/geronb/gbac070 35642746 PMC9535783

[CIT0004] Börsch-Supan, A., Brandt, M., Hunkler, C., Kneip, T., Korbmacher, J., Malter, F., Schaan, B., Stuck, S., & Zuber, S. (2013). Data resource profile: The Survey of Health, Ageing and Retirement in Europe (SHARE). International Journal of Epidemiology, 42(4), 992–1001. 10.1093/ije/dyt088 23778574 PMC3780997

[CIT0005] Cambois, E., Blachier, A., & Robine, J.-M. (2013). Aging and health in France: An unexpected expansion of disability in mid-adulthood over recent years. European Journal of Public Health, 23(4), 575–581. 10.1093/eurpub/cks136 23042230

[CIT0006] Caplan, Z., & Rabe, M. (2023). *The older population: 2020* (C2020BR-07; 2020 census briefs). United States Census Bureau.

[CIT0007] Chang, V. W., Alley, D. E., & Dowd, J. B. (2017). Trends in the relationship between obesity and disability, 1988–2012. *American Journal of Epidemiology*, 186(6), 688–695. 10.1093/aje/kwx092 28486588

[CIT0008] Chatterji, S., Byles, J., Cutler, D., Seeman, T., & Verdes, E. (2015). Health, functioning, and disability in older adults—Present status and future implications. Lancet (London, England), 385(9967), 563–575. 10.1016/S0140-6736(14)61462-8 25468158 PMC4882096

[CIT0009] Chen, Y., Wilkens, J., Shao, K., Rebellato, G., Phillips, D., & Lee, J. (2022). Harmonised SHARE documentation. Version F (2004–2020). USC Dornsife Center for Economic and Social Research, Program on Global Aging, Health and Policy.

[CIT0010] Córdoba-Doña, J. A., Benítez-Rodríguez, E., Escolar-Pujolar, A., & Santos-Sánchez, V. (2023). Age-period-cohort analysis of lung cancer mortality inequalities in Southern Spain: Missed opportunities for implementing equitable tobacco control policies. International Journal for Equity in Health, 22(1), 132. 10.1186/s12939-023-01946-y 37438851 PMC10339480

[CIT0011] Dodds, R., Kuh, D., Aihie Sayer, A., & Cooper, R. (2013). Physical activity levels across adult life and grip strength in early old age: Updating findings from a British birth cohort. Age and Ageing, 42(6), 794–798. 10.1093/ageing/aft124 23981980 PMC3809720

[CIT0012] Eurostat. (2023, February). Population structure and ageing. Eurostat. https://ec.europa.eu/eurostat/statistics-explained/index.php?title=Population_structure_and_ageing

[CIT0013] Fors, S., Illinca, S., Jull, J., Kadi, S., Phillips, S. P., Rodrigues, R., Vafaei, A., Zolyomi, E., & Rehnberg, J. (2022). Cohort-specific disability trajectories among older women and men in Europe 2004–2017. European Journal of Ageing, 19(4), 1111–1119. 10.1007/s10433-022-00684-4 36506653 PMC9729672

[CIT0014] Freedman, V. A., Spillman, B. C., Andreski, P. M., Cornman, J. C., Crimmins, E. M., Kramarow, E., Lubitz, J., Martin, L. G., Merkin, S. S., Schoeni, R. F., Seeman, T. E., & Waidmann, T. A. (2013). Trends in late-life activity limitations in the United States: An update from five national surveys. Demography, 50(2), 661–671. 10.1007/s13524-012-0167-z 23104207 PMC3586750

[CIT0015] Fries, J. F. (1980). Aging, natural death, and the compression of morbidity. The New England Journal of Medicine, 303(3), 130–135. 10.1056/NEJM198007173030304 7383070

[CIT0016] Garcia, J., & Quintana-Domeque, C. (2007). The evolution of adult height in Europe: A brief note. Economics and Human Biology, 5(2), 340–349. 10.1016/j.ehb.2007.02.002 17412655

[CIT0017] Gondek, D., Bann, D., Ning, K., Grundy, E., & Ploubidis, G. B. (2019). Post-war (1946–2017) population health change in the United Kingdom: A systematic review. PLoS One, 14(7), e0218991. 10.1371/journal.pone.0218991 31269039 PMC6608959

[CIT0018] Gondek, D., Bann, D., Patalay, P., Goodman, A., McElroy, E., Richards, M., & Ploubidis, G. B. (2020). Psychological distress from early adulthood to early old age: Evidence from the 1946, 1958 and 1970 British birth cohorts. Psychological Medicine, 52, 1471–1480. 10.1017/s003329172000327x PMC9226427 33472020

[CIT0019] Gondek, D., Ploubidis, G. B., Hossin, M. Z., Gao, M., Bann, D., & Koupil, I. (2021). Inequality in hospitalization due to non-communicable diseases in Sweden: Age-cohort analysis of the Uppsala Birth Cohort Multigenerational Study. SSM—Population Health, 13, 100741. 10.1016/j.ssmph.2021.100741 33537404 PMC7841359

[CIT0020] Hiam, L., Harrison, D., McKee, M., & Dorling, D. (2018). Why is life expectancy in England and Wales ‘stalling’? Journal of Epidemiology & Community Health, 72(5), 404–408. 10.1136/jech-2017-210401 29463599

[CIT0021] Jivraj, S., Goodman, A., Pongiglione, B., & Ploubidis, G. B. (2020). Living longer but not necessarily healthier: The joint progress of health and mortality in the working-age population of England. Population Studies, 74(3), 399–414. 10.1080/00324728.2020.1767297 32659174

[CIT0022] Johnson, W., Li, L., Kuh, D., & Hardy, R. (2015). How has the age-related process of overweight or obesity development changed over time? Co-ordinated analyses of individual participant data from five United Kingdom birth cohorts. PLoS Medicine, 12(5), e1001828. 10.1371/journal.pmed.1001828 25993005 PMC4437909

[CIT0023] Koivunen, K., Sillanpää, E., Munukka, M., Portegijs, E., & Rantanen, T. (2021). Cohort differences in maximal physical performance: A comparison of 75- and 80-year-old men and women born 28 years apart. The Journals of Gerontology, Series A: Biological Sciences and Medical Sciences, 76(7), 1251–1259. 10.1093/gerona/glaa224 32886740

[CIT0024] Kuh, D., Hardy, R., Butterworth, S., Okell, L., Wadsworth, M., Cooper, C., & Aihie Sayer, A. (2006). Developmental origins of midlife grip strength: Findings from a birth cohort study. The Journals of Gerontology, Series A: Biological Sciences and Medical Sciences, 61(7), 702–706. 10.1093/gerona/61.7.702 16870632

[CIT0025] Lafortune, G., & Balestat, G. (2007). Trends in severe disability among elderly people: Assessing the evidence in 12 OECD countries and the future implications. OECD. 10.1787/217072070078

[CIT0026] Lee, J., Lau, S., Meijer, E., & Hu, P. (2020). Living longer, with or without disability? A global and longitudinal perspective. The Journals of Gerontology, Series A: Biological Sciences and Medical Sciences, 75(1), 162–167. 10.1093/gerona/glz007 30629214 PMC6909890

[CIT0027] Martin, L. G., Freedman, V. A., Schoeni, R. F., & Andreski, P. M. (2010). Trends in disability and related chronic conditions among people ages fifty to sixty-four. Health Affairs (Project Hope), 29(4), 725–731. 10.1377/hlthaff.2008.0746 20368601 PMC2874878

[CIT0028] Martinson, M. L., Lapham, J., Ercin-Swearinger, H., Teitler, J. O., & Reichman, N. E. (2022). Generational shifts in young adult cardiovascular health? Millennials and Generation X in the United States and England. The Journals of Gerontology, Series B: Psychological Sciences and Social Sciences, 77(Supplement_2), S177–S188. 10.1093/geronb/gbac036 35195713 PMC9154229

[CIT0029] Masters, R. K., Tilstra, A. M., & Simon, D. H. (2018). Explaining recent mortality trends among younger and middle-aged White Americans. International Journal of Epidemiology, 47(1), 81–88. 10.1093/ije/dyx127 29040539 PMC6658718

[CIT0030] McKeown, T., & Record, R. G. (1962). Reasons for the decline of mortality in England and Wales during the nineteenth century. Population Studies, 16(2), 94–122. 10.2307/2173119 11630508

[CIT0031] Moe, J. O., & Hagen, T. P. (2011). Trends and variation in mild disability and functional limitations among older adults in Norway, 1986–2008. European Journal of Ageing, 8(1), 49–61. 10.1007/s10433-011-0179-3 21475398 PMC3047681

[CIT0032] ONS. (2023). Profile of the older population living in England and Wales in 2021 and changes since 2011. Office for National Statistics. https://www.ons.gov.uk/peoplepopulationandcommunity/birthsdeathsandmarriages/ageing/articles/profileoftheolderpopulationlivinginenglandandwalesin2021andchangessince2011/2023-04-03

[CIT0033] Oostrom, S. H. van, Gijsen, R., Stirbu, I., Korevaar, J. C., Schellevis, F. G., Picavet, H. S. J., & Hoeymans, N. (2016). Time trends in prevalence of chronic diseases and multimorbidity not only due to aging: Data from general practices and health surveys. PLoS One, 11(8), e0160264. 10.1371/journal.pone.0160264 27482903 PMC4970764

[CIT0034] Pang, M., Kaufman, J. S., & Platt, R. W. (2016). Studying noncollapsibility of the odds ratio with marginal structural and logistic regression models. Statistical Methods in Medical Research, 25(5), 1925–1937. 10.1177/0962280213505804 24108272

[CIT0035] Parker, M. G., & Thorslund, M. (2007). Health trends in the elderly population: Getting better and getting worse. Gerontologist, 47(2), 150–158. 10.1093/geront/47.2.150 17440120

[CIT0036] Payne, C. F. (2022). Expansion, compression, neither, both? Divergent patterns in healthy, disability-free, and morbidity-free life expectancy across U.S. birth cohorts, 1998–2016. Demography, 59, 949–973. 10.1215/00703370-9938662 35522071

[CIT0037] Ploubidis, G. B., & Grundy, E. (2011). Health measurement in population surveys: Combining information from self-reported and observer-measured health indicators. Demography, 48(2), 699–724. 10.1007/s13524-011-0028-1 21506021

[CIT0038] Ploubidis, G. B., & Pongiglione, B. (2019). Self-rated health across the life course: Evidence from the 1958 and 1970 British birth cohorts. In G. B. Ploubidis, B. Pongiglione, B. De Stavola, R. Daniel, L. Benova, E. Grundy, & S. Read (Eds.), Pathways to health (pp. 79–97). Springer.

[CIT0039] Preston, S. H., & Wang, H. (2006). Sex mortality differences in the United States: The role of cohort smoking patterns. Demography, 43(4), 631–646. 10.1353/dem.2006.0037 17236538

[CIT0040] Reynolds, S. L., Crimmins, E. M., & Saito, Y. (1998). Cohort differences in disability and disease presence. Gerontologist, 38(5), 578–590. 10.1093/geront/38.5.578 9803646

[CIT0041] Scott, A. J. (2021). The longevity society. The Lancet Healthy Longevity, 2(12), e820–e827. 10.1016/S2666-7568(21)00247-6 36098038

[CIT0042] Seeman, T. E., Merkin, S. S., Crimmins, E. M., & Karlamangla, A. S. (2010). Disability trends among older Americans: National Health and Nutrition Examination Surveys, 1988–1994 and 1999–2004. American Journal of Public Health, 100(1), 100–107. 10.2105/AJPH.2008.157388 19910350 PMC2791257

[CIT0043] Sonnega, A., Faul, J. D., Ofstedal, M. B., Langa, K. M., Phillips, J. W., & Weir, D. R. (2014). Cohort profile: The Health and Retirement Study (HRS). International Journal of Epidemiology, 43(2), 576–585. 10.1093/ije/dyu067 24671021 PMC3997380

[CIT0044] Soysal, P., Hurst, C., Demurtas, J., Firth, J., Howden, R., Yang, L., Tully, M. A., Koyanagi, A., Ilie, P. C., López-Sánchez, G. F., Schwingshackl, L., Veronese, N., & Smith, L. (2021). Handgrip strength and health outcomes: Umbrella review of systematic reviews with meta-analyses of observational studies. Journal of Sport and Health Science, 10(3), 290–295. 10.1016/j.jshs.2020.06.009 32565244 PMC8167328

[CIT0045] Sperlich, S., Beller, J., Epping, J., Safieddine, B., Tetzlaff, J., & Geyer, S. (2021). Are disability rates among people with diabetes increasing in Germany? A decomposition analysis of temporal change between 2004 and 2015. Journal of Aging and Health, 33(3–4), 205–216. 10.1177/0898264320970324 33135530 PMC7917560

[CIT0046] Spiers, G. F., Kunonga, T. P., Beyer, F., Craig, D., Hanratty, B., & Jagger, C. (2021). Trends in health expectancies: A systematic review of international evidence. BMJ Open, 11(5), e045567. 10.1136/bmjopen-2020-045567 PMC8154999 34035101

[CIT0047] Steptoe, A., Breeze, E., Banks, J., & Nazroo, J. (2013). Cohort profile: The English Longitudinal Study of Ageing. International Journal of Epidemiology, 42(6), 1640–1648. 10.1093/ije/dys168 23143611 PMC3900867

[CIT0048] UN. (2020). World population prospects 2022: Summary of results. United Nations Department of Economic and Social Affairs, Population Division. https://www.un.org/development/desa/pd/sites/www.un.org.development.desa.pd/files/wpp2022_summary_of_results.pdf

[CIT0049] Walter, S., Beltrán-Sánchez, H., Regidor, E., Gomez-Martin, C., del-Barrio, J. L., Gil-de-Miguel, A., Subramanian, S. V., & Gil-Prieto, R. (2016). No evidence of morbidity compression in Spain: A time series study based on national hospitalization records. International Journal of Public Health, 61(7), 729–738. 10.1007/s00038-016-0829-5 27233641 PMC7446746

[CIT0050] Welsh, C. E., Matthews, F. E., & Jagger, C. (2021). Trends in life expectancy and healthy life years at birth and age 65 in the UK, 2008–2016, and other countries of the EU28: An observational cross-sectional study. The Lancet Regional Health—Europe, 2, 100023. 10.1016/j.lanepe.2020.100023 33870247 PMC8042672

[CIT0051] Wilkens, J., Green, H., Petrosyan, S., Rebellato, G., Phillips, D., & Lee, J. (2022). Harmonised HRS documentation. Version C (1992–2019). USC Dornsife Center for Economic and Social Research, Program on Global Aging, Health and Policy.

[CIT0052] Wilkens, J., Rebellato, G., Oh, Y., & Lee, J. (2021). Harmonised ELSA documentation. Version G.2 (2002–2019). USC Dornsife Center for Economic and Social Research, Program on Global Aging, Health and Policy.

[CIT0053] Zheng, H., Dirlam, J., Choi, Y., & George, L. (2023). Understanding the health decline of Americans in boomers to millennials. Social Science & Medicine, 337, 116282. 10.1016/j.socscimed.2023.116282 37832317

[CIT0054] Zheng, H., & Echave, P. (2021). Are recent cohorts getting worse? Trends in US adult physiological status, mental health, and health behaviors across a century of birth cohorts. American Journal of Epidemiology, 190(11), 2242–2255. 10.1093/aje/kwab076 33738469 PMC8799895

[CIT0055] Zou, G. (2004). A modified Poisson regression approach to prospective studies with binary data. American Journal of Epidemiology, 159(7), 702–706. 10.1093/aje/kwh090 15033648

[CIT0056] Zou, G., & Donner, A. (2013). Extension of the modified Poisson regression model to prospective studies with correlated binary data. Statistical Methods in Medical Research, 22(6), 661–670. 10.1177/0962280211427759 22072596

[CIT0057] Zunzunegui, M. V., Nunez, O., Durban, M., García de Yébenes, M. J., & Otero, A. (2006). Decreasing prevalence of disability in activities of daily living, functional limitations and poor self-rated health: A 6-year follow-up study in Spain. Aging Clinical and Experimental Research, 18(5), 352–358. 10.1007/BF03324830 17167298

